# Endoscopic therapy for recurrent pancreatitis complicated with pancreatolithiasis in a case of annular pancreas

**DOI:** 10.1002/deo2.122

**Published:** 2022-04-21

**Authors:** Saori Mizutani, Naoki Okano, Hiroki Nakagawa, Koji Watanabe, Yuto Yamada, Yusuke Kimura, Kensuke Yoshimoto, Susumu Iwasaki, Seiichi Hara, Kensuke Takuma, Yui Kishimoto, Ken Ito, Takahisa Matsuda, Yoshinori Igarashi

**Affiliations:** ^1^ Division of Gastroenterology and Hepatology Toho University Omori Medical Center Tokyo Japan

**Keywords:** annular pancreas, endoscopic pancreatic sphincterotomy, pancreatitis of annular pancreas

## Abstract

Annular pancreas is a congenital abnormality in which part of the pancreatic head completely or partially surrounds the duodenum in a ring‐like manner. The condition is thought to be an abnormality of the ventral pancreatic bud. While pancreatitis is a common complication of the annular pancreas, its recurrence may be prevented by improving the outflow of pancreatic juice. The present case report describes a 23‐year‐old woman who had been referred to our hospital for recurrent pancreatitis since childhood. An endoscopic incision was made on the orifice of the annular pancreas, after which pancreatitis of the annular pancreas did not recur for 6 years. The patient subsequently exhibited pancreatolithiasis in the dorsal pancreatic duct, which was successfully treated with endoscopic treatment. Endoscopic pancreatic sphincterotomy may prevent the recurrence of pancreatitis and avoid further surgical interventions by improving the flow of pancreatic juice.

## INTRODUCTION

Annular pancreas is a congenital anomaly in which a part of the pancreatic head surrounds the duodenum in a ring‐like manner. It is believed to be the result of malrotation of the ventral pancreatic bud. The condition has been reported in three of 20,000 autopsy cases,^1^ and although it was once considered an extremely rare disease, the number of reports has increased because endoscopic retrograde cholangiopancreatography (ERCP) has become more widely performed. Common symptoms in adults include pain, vomiting, gastroduodenal ulcer, and pancreatitis. Here, we report a case of the annular pancreas in which endoscopic therapy improved the symptoms of recurrent pancreatitis complicated with pancreatolithiasis.

## CASE REPORT

The case was a 23‐year‐old woman who was diagnosed with annular pancreas at another hospital after developing acute pancreatitis at the age of 12 years. The patient subsequently experienced repeated episodes of acute pancreatitis, and after being treated at a previous hospital for acute pancreatitis at the age of 23 years, the patient was given an initial referral to our hospital. The case involved no history of alcohol consumption or smoking.

During the examination at our hospital, the patient's vital signs and abdominal findings were both normal. A blood test showed slightly elevated lipase levels at 71 U/L.

**FIGURE 1:**
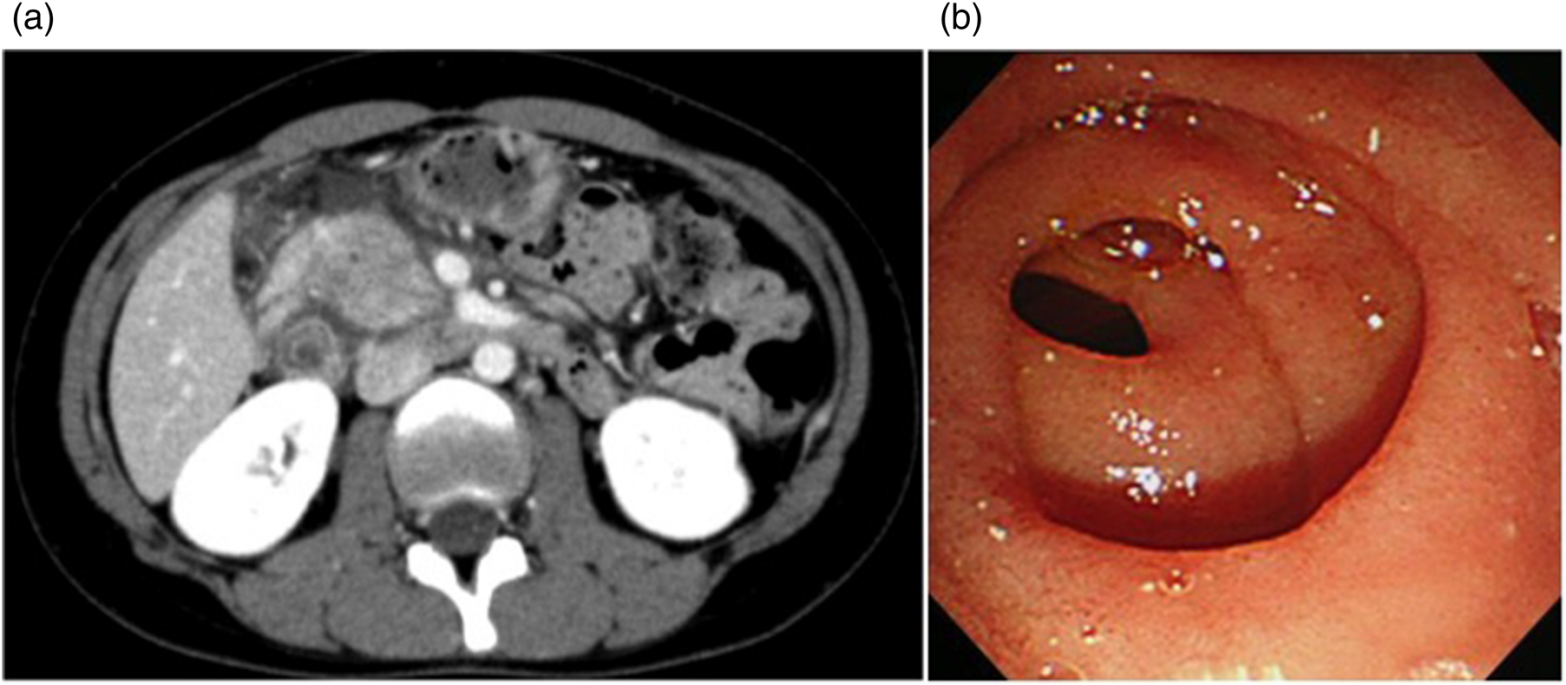
(a) Abdominal computed tomography showed acute pancreatitis localized to the annular pancreas. (b) Endoscopy revealed ring‐shaped stenosis in the descending part of the duodenum.

Contrast‐enhanced computed tomography for acute pancreatitis that had been performed at the previous hospital showed pancreatic enlargement centered around the pancreatic head and increased enhancement of the surrounding adipose tissue, which indicated acute pancreatitis localized to the annular pancreas (Figure 1a).

Magnetic resonance cholangiopancreatography depicted the pathways of the pancreatic duct in a ring shape in the pancreatic head, dorsal pancreatic duct, and common bile duct. Endoscopy revealed ring‐shaped stenosis in the descending part of the duodenum (Figure 1b.). In the papilla, the bile duct and pancreatic duct appeared as separated orifices.

**FIGURE 2:**
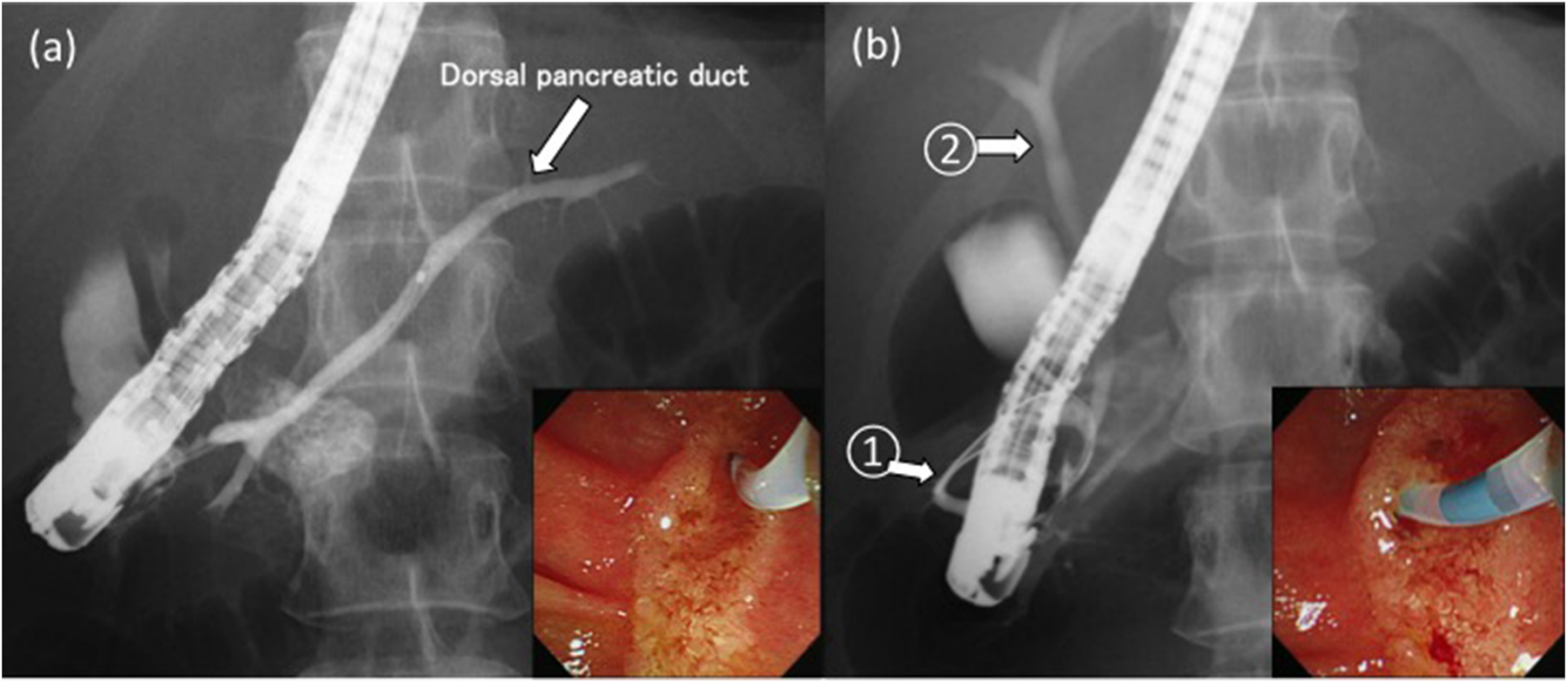
(a) Endoscopic retrograde cholangiopancreatography contrast imaging from the upper right orifice revealed the dorsal pancreatic duct and a part of the annular pancreatic duct. (b) Endoscopic retrograde cholangiopancreatography contrast imaging from the left orifice showed the annular pancreatic duct (1) and common bile duct (2).

With ERCP, contrast imaging from the upper‐right orifice revealed the dorsal pancreatic duct and a part of the annular duct (Figure 2a). Contrast imaging from the left orifice showed the annular duct and common bile duct (Figure 2b). Because pancreatitis of the annular pancreas was observed, endoscopic pancreatic sphincterotomy (EPST) was performed on the left orifice (which was considered to be the main duct of the annular pancreas) to promote the outflow of pancreatic juice from the annular pancreas. For 6 years following this procedure, there was no recurrence of pancreatitis.

**FIGURE 3:**
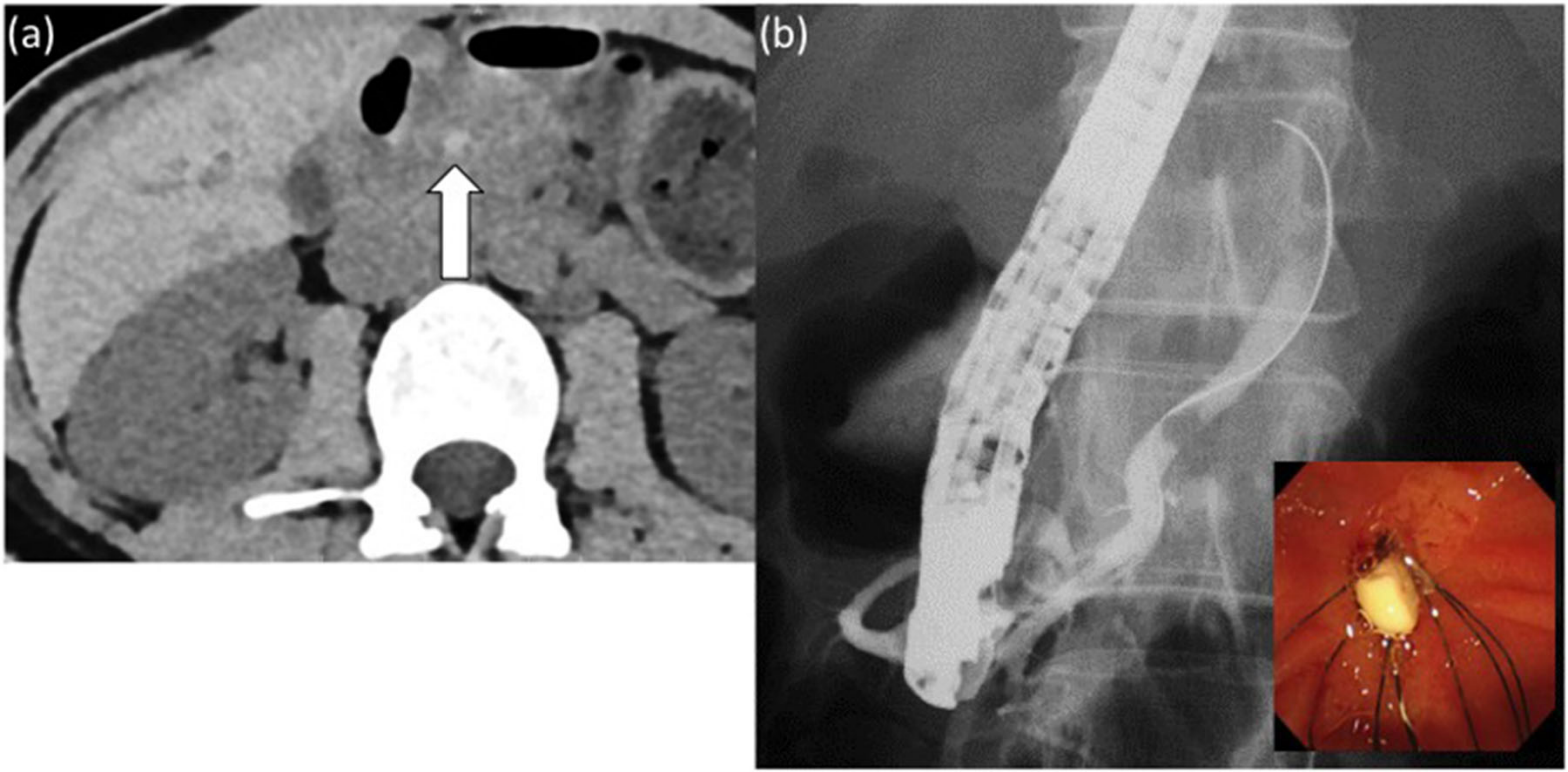
(a) A high‐density lesion was identified (arrow), which was thought to be a stone in the dorsal pancreatic duct. (b) endoscopic retrograde cholangiopancreatography showed a circular translucency image in the dorsal pancreatic duct. Endoscopic pancreatic sphincterotomy was performed on the dorsal pancreatic duct and pancreatic calculi were removed with a basket catheter.

The patient once again developed acute pancreatitis at the age of 29 years and was referred to our hospital for the second time. Abdominal computed tomography showed acute pancreatitis in the pancreatic body and tail, with a high‐density lesion which was thought to be a stone in the dorsal pancreatic duct (Figure 3a). Contrast imaging from the orifice of the dorsal pancreatic duct with ERCP showed a 5‐mm circular translucency image in the dorsal pancreatic duct. EPST was performed on the dorsal pancreatic duct and pancreatic calculi were removed with a basket catheter (Figure 3b). The patient's course was favorable, and she was discharged from the hospital. In the subsequent 18 months of outpatient care, the patient did not exhibit recurrence of pancreatitis.

## DISCUSSION

The annular pancreas is considered to result from the abnormal development of the ventral pancreatic bud. Usually, during the first 4–8 weeks of embryonic development, the dorsal and ventral pancreatic buds rotate and fuse as a result of dilatation of the duodenum.^2^

**FIGURE 4:**
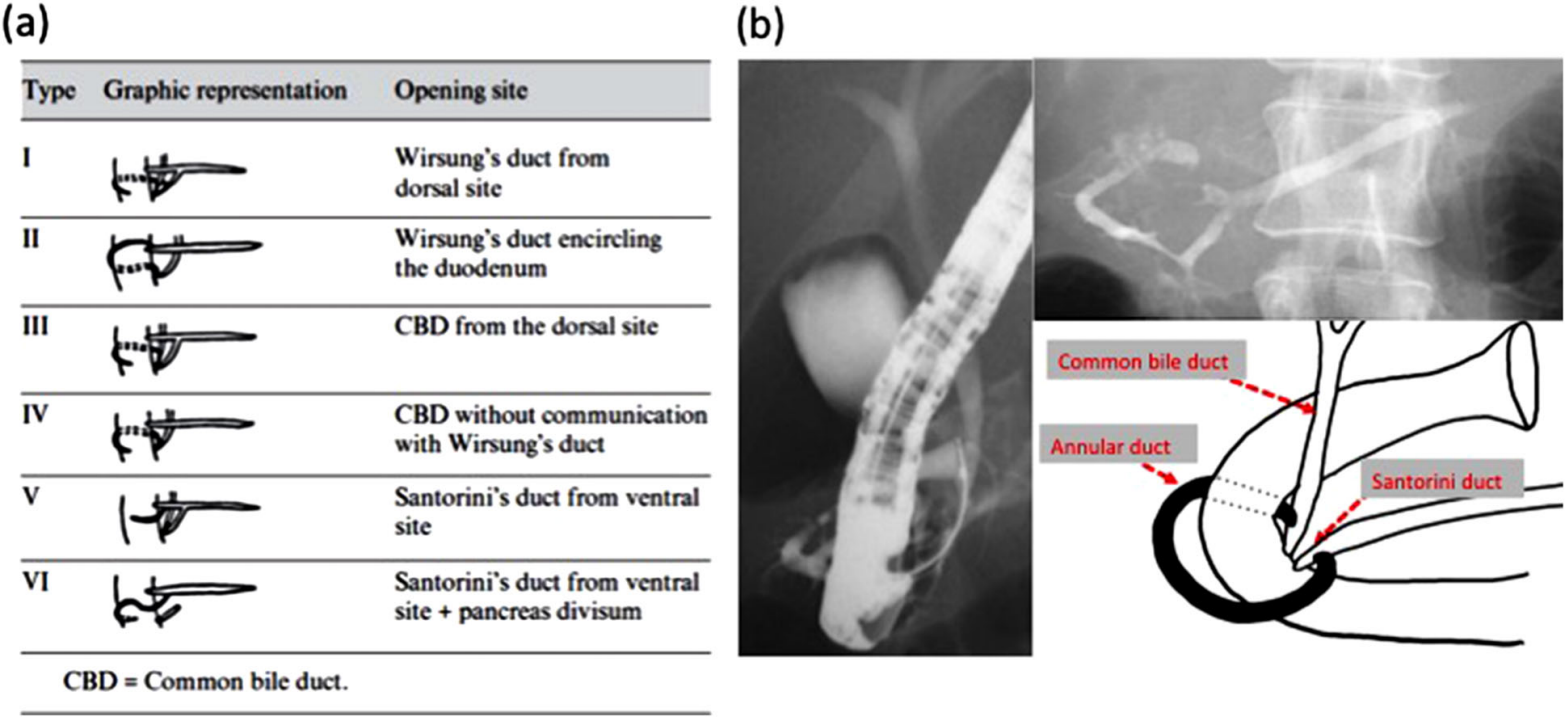
(a) Yogi et al. described six types of annular pancreas according to the outflow side of the annular pancreatic duct. (b) In the present case, endoscopic retrograde cholangiopancreatography showed that the annular pancreatic duct was connected to the common bile duct and duct of Santorini.

Yogi et al. classified the annular pancreas into six types, depending on the site of the annular duct (Figure 4a).^3^ The most common was type I, in which the annular duct flows directly into the main pancreatic duct. The second most common was type II, in which the Wirsung duct encircles the duodenum and outflow is at the major duodenal papilla. The other four types of annular duct were much less common.

In the present case, ERCP showed that the annular duct was connected to the common bile duct and Santorini duct, which is type II under the classification system of Yogi et al. (Figure 4b), and complicated with the anomalous connection of pancreatobiliary ducts.

The annular pancreas is definitively diagnosed when the annular duct is identified by ERCP or magnetic resonance cholangiopancreatography. In duodenography, ring‐shaped stenosis is often observed in the descending part of the duodenum. In computed tomography, the pancreatic parenchyma is contiguous with the pancreatic head surrounding the descending part of the duodenum.

Symptoms of the annular pancreas vary greatly depending on the timing of their onset. In the neonatal stage, the condition is associated with congenital duodenal obstruction, and many cases present with symptoms of duodenal stenosis, such as frequent vomiting.

Some adult cases are asymptomatic; however, symptoms include upper abdominal pain, nausea, vomiting, abdominal distention, jaundice, and weight loss caused by duodenal stenosis or complications such as peptic ulcer and cholelithiasis. The three main complications of the annular pancreas are peptic ulcers, cholelithiasis, and pancreatitis, which according to Yogi et al., occur in 25%, 16%, and 11% of cases, respectively.^3^

Treatment of the annular pancreas is tailored to the symptoms that appear in each case. Asymptomatic cases do not require treatment. Internal medical treatment is the first choice for upper abdominal discomfort, mild pancreatitis, and gastric or duodenal ulcers caused by mild duodenal stenosis. Persistent duodenal obstruction can be treated by bypassing the annular part with gastrojejunostomy or duodenojejunostomy.^4^ Pancreatoduodenectomy (Whipple procedure) can be considered for treatment in patients with complications such as chronic annular pancreatitis, pancreatolithiasis in the annular pancreas, or suspected coexistence of malignant tumors.^5^

My research in PubMed with the keyword “annular pancreas” and “pancreatolithiasis” revealed that there were five cases of pancreatolithiasis in the annular pancreas reported in Japan by 1999. Surgery was recommended for repeated cases of pancreatitis and there were no reports of successful endoscopic treatment in Japan.^6^ A previous case report in 2020 from India reported that a case of repeated recurrent acute pancreatitis in the annular pancreas was performed for an endoscopic pancreatic duct sphincterotomy treatment and it was effective.^7^ But this case was not complicated with pancreatolithiasis.

The present case exhibited recurrent pancreatitis complicated with pancreatolithiasis in the annular pancreas. The initial incision of the annular duct orifice prevented pancreatitis for 6 years. Subsequently, there was a recurrence of pancreatitis complicated with pancreatolithiasis in the dorsal pancreatic duct. Endoscopic incision of the dorsal pancreatic duct orifice and removal of the pancreatic calculi successfully prevented the recurrence of pancreatitis.

The pancreatitis of the annular pancreas is generally confined to the annual pancreas, and its pathogenesis is probably related to the impairment of pancreatic secretion flow through the annular duct. In this case, the annular pancreas is complicated by the anomalous connection of pancreatobiliary ducts. Therefore, the reason why pancreatolithiasis was observed in the dorsal pancreatic duct may result from the impairment of pancreatic secretion flow through the dorsal pancreatic duct. As a result, EPST for both the orifices of the pancreatic duct might be more effective for recurrent pancreatitis in the annular pancreas complicated by the anomalous connection of pancreatobiliary ducts. Although no long‐term follow‐up was conducted, and EPST is not established as a method for preventing the recurrence of acute pancreatitis in the annular pancreas, EPST may prevent the recurrence of pancreatitis and further surgical interventions by improving the flow of pancreatic juice.

## CONFLICT OF INTEREST

The authors have no conflict of interest to declare.

## FUNDING INFORMATION

None.

## ETHICS STATEMENT

This case report was performed in accordance with the World Medical Association Declaration of Helsinki.
